# Critical survival periods in prostate cancer in Sweden explored by conditional survival analysis

**DOI:** 10.1002/cam4.7126

**Published:** 2024-03-28

**Authors:** Kari Hemminki, Frantisek Zitricky, Kristina Sundquist, Jan Sundquist, Asta Försti, Akseli Hemminki, Otto Hemminki

**Affiliations:** ^1^ Biomedical Center, Faculty of Medicine Charles University Pilsen Pilsen Czech Republic; ^2^ Division of Cancer Epidemiology, German Cancer Research Center (DKFZ) Heidelberg Germany; ^3^ Center for Primary Health Care Research Lund University Malmö Sweden; ^4^ Department of Family Medicine and Community Health, Department of Population Health Science and Policy Icahn School of Medicine at Mount Sinai New York City New York USA; ^5^ Department of Functional Pathology, School of Medicine, Center for Community‐based Healthcare Research and Education (CoHRE) Shimane University Izumo Japan; ^6^ Hopp Children's Cancer Center (KiTZ) Heidelberg Germany; ^7^ Division of Pediatric Neurooncology, German Cancer Research Center (DKFZ) German Cancer Consortium (DKTK) Heidelberg Germany; ^8^ Cancer Gene Therapy Group, Translational Immunology Research Program University of Helsinki Helsinki Finland; ^9^ Comprehensive Cancer Center Helsinki University Hospital Helsinki Finland; ^10^ Department of Urology Helsinki University Hospital Helsinki Finland

**Keywords:** age, conditional relative survival, prognosis, stage, treatment

## Abstract

**Backround:**

We wanted to characterize conditional survival in prostate cancer (PC) in Sweden around and after 2005 when the vast increase in incidence due to the opportunistic testing for prostate specific antigen (PSA) culminated. We hypothesize that analyzing survival data during that time period may help interpret survival trends. We focus on stage‐specific analysis using conditional survival in order to define the periods when deaths most commonly occurred.

**Methods:**

Data on PC patients were obtained from the Swedish cancer registry for analysis of 1‐, 2.5‐ and 5‐year relative survival and conditional relative survival between years 2004 and 2018. Tumor‐node‐metastatic stage classification at diagnosis was used to specify survival.

**Results:**

Small improvements were observed in stage‐ and age‐related relative survival duriring the study period. Applying conditional relative survival showed that survival in stage T3 up to 2.5 years was better than survival between years 2.5 and 5. Survival in stage T4 was approximately equal in the first and the subsequent 2.5‐year period. For M1, the first 2.5 year survival period was worse than the subsequent one. The proportion of high risk and M1 disease in old patients (80+ years) remained very high and their survival improved only modestly.

**Conclusions:**

The data indicate that M1 metastases kill more patients in the first 2.5 years than between years 2.5 and 5 after diagnosis; T4 deaths are equal in the two periods, and in T3 mortality in the first 2.5‐year period is lower than between years 2.5 and 5 after diagnosis. Conditional survival could be applied to explore critical survival periods even past 5 years after diagnoses and to monitor success in novel diagnostic and treatment practices. Improvement of survival in elderly patients may require clinical input.

## 1 INTRODUCTION

Opportunistic (non‐organized) testing for prostate specific antigen (PSA) has led to a large increase in the incidence of prostate cancer (PC) in many countries starting in the late 1980s. In Sweden and in the neighboring countries the increase in incidence leveled off at around 2005 with a subsequent slow decrease.^1^ Presentation of PC in the PSA era (when PSA testing has been available) has featured lower diagnostic age, less advanced tumor characteristics (lower tumor T stage, according to the clinical tumor‐node‐metastasis, TNM system) and lower proportion of patients presenting with distant metastases.^2^ While survival in PC has constantly improved through the PSA era, difficulties have remained in assigning the positive development to earlier stages or age at diagnosis, improvements in treatment or overdiagnosis.^2^, ^3^, ^4^, ^5^, ^6^ After culmination of the incidence it may be assumed that the proportion of overdiagnosed patients has stabilized, and as patient age and stage (in Sweden TNM classification was complete since 2003) can be controlled, it is likely that the effect of treatment can be more reliably estimated even though no individual data are available on treatment.^7^

In Sweden, the distribution of risk classes according to the national quality register for PC in 2003 was 23.8% low risk (T1‐2, Gleason ≤6, PSA < 10 ng/mL), 23.8% intermediate risk (T1‐2, Gleason 7 and/or PSA 10 to <20 ng/mL), 25.7% high risk (T3 and/or Gleason 8–10 and/or PSA 20 to <50 ng/mL), 8.7% local metastases (T4 and/or N1 and/or PSA 50 to <100 ng/mL), 15.9% distant metastases (M1 and/or PSA ≥100 ng/mL) and 2.1% missing (https://statistik.incanet.se/npcr/). In 2018 the class distribution was (%) 23.6, 37.1, 19.3, 5.8, 11.6, and missing 2.6, thus showing an increase in the proportion of intermediate risk and decrease in all higher risk categories. The most recent data are for 2022, when the intermediate risk proportion was 40.3% and the high‐risk proportion increased to 22.4%. The current 5‐year relative survival in PC in Sweden is about 95%, which indicates that only 5% of those who died of PC were diagnosed in the past 5 years and that the large majority of them were diagnosed a long time ago.^8^

Among Swedish men who died of PC between 1992 and 2016, the proportion of localized disease at primary diagnosis increased from 34% to 48%, while the proportion of distant metastases at diagnosis decreased from 56% to 42%.^9^ Median PC disease duration (diagnosis to death in PC) increased from 3.3 to 5.9 years. For Swedish men who died between years 1987 and 2006 and had a PC diagnosis, 50% died of PC while the underlying cause of death for the other 50% was a non‐PC cause.^10^ Similarly, in Denmark between 2007 and 2016 the underlying cause of death in PC patients was PC for 53% of men.^11^ Among men who died of PC, 35.5% were diagnosed with localized, 29.0% with locally advanced and 35.5% with M1 PC; among men with other causes of death the proportions were 65.1%, 22.6%, and 12.3%.^11^

The traditional treatment for metastatic PC has been androgen deprivation therapy (ADT) and for castration‐resistant tumors several new therapeutic options have become available from about 2010 to 2020 as discussed in terms of the national treatment guidelines.^12^ The novel agents include docetaxel, abiraterone, enzalutamide, cabazitaxel, sipuleucel‐T, radium‐223, and others but as the present follow‐up terminated in 2018 the novel therapies may impact survival only towards the end of the study or not at all.^13^, ^14^, ^15^, ^16^, ^17^ In Sweden 83% of patients diagnosed with locally advanced tumors survived 5 years in 2008–11^18^; for metastatic patients the median survival in 2010–15 was 2.7 years.^13^ Some 15% of patients with locally advanced tumors received radical treatment in year 2000 but the percentage increased to over 40% in 15 years; radical radiotherapy (with ADT) was more commonly applied than radical prostatectomy, for which robotic surgery was introduced after year 2000.^15^, ^18^

We assess here stage‐ and age‐specific relative survival and conditional relative survival in PC in Sweden from 2004 to 2017 and correlate the results to diagnostic and treatment data available in the publically available national quality register for PC through these years. Conditional survival enables a better definition of critical survival periods after diagnoses up to 5 years.^19^ In reference to the above discussion about cause‐specific and overall deaths, relative survival considers all deaths in PC patients.

## 2 METHODS

Cancer data were obtained from the Swedish population‐based cancer registry from years 2004 through 2018. The population of Sweden was obtained from the total population register. The linkages between the different registers were done using the personal identification number, assigned to each resident in Sweden, and replaced by a serial number to preserve people's integrity. Cases were included when they had specified data on T (0–4 or x) and M stages and when patients had a residence status in Sweden at the time of diagnosis. A total of 42,402 PC cases were included for 2004–08 and 46,649 PC cases were included for 2009–13. The respective case numbers in the cancer registry were 47,339 and 49,330. For 1‐year survival the last covered year was 2017, and for 5‐year survival it was 2013.

Data analysis was performed in SAS and R, using custom‐made scripts in which cumulative excess hazard was calculated for pre‐defined intervals of follow‐up, as described.^20^ The approach allows to consider person‐time at resolution of days, while maintaining computational efficiency. Relative survival was calculated using an adopted version of the Pohar Perme estimator which is particularly suitable for cpmparisons of survival in periods or populations.^21^, ^22^ The follow‐up period was divided into 5 days following date of diagnosis and subsequent 10‐day long intervals. The expected mortality was calculated based on age and calendar year, using population lifetables.^23^ Patients were treated as censored at the date of first emigration after diagnosis.

We calculated relative survival using custom‐made code, using approach similar to (Coviello et al., 2015), where cumulative excess hazard is calculated for pre‐defined intervals of follow‐up. The approach allows to consider person‐time at resolution of days, while maintaining computational efficiency.

Conditional relative survival was calculated by dividing the respective age‐standardized relative survival estimates. In conditional 5/1‐year survival patients were followed from years 1 to 5; in conditional 5/2.5‐year survival patients were followed from years 2.5 to 5. Confidence intervals (95%) of all estimators were derived by approximating variance on a logarithmic scale using the delta method.^24^ The 95%‐confidence intervals bounds are given by:

$$CI_{95\%}=\exp\;\left(\log S\pm q_{0.975}\sqrt{\mathrm{Var}\left(\log\left(S\right)\right)}\;\right)$$

where *S* is the estimated relative survival and $q_{0.975}$ is the 0.975 quantile of the standard normal distribution. The square root of $\mathrm{Var}\left(\log\left(S\right)\right)$ corresponds to the standard error of the cumulative hazard.

The temporal trends in relative survival were calculated by grouping patients according to diagnosis date into 2.5‐year periods, with an exception of 1‐year age‐specific relative survival, where the most recent period covered 1.5 years. To display temporal trends in relative survival, each estimate was assigned a timepoint in the middle of the respective 2.5‐year or 1.5‐year periods. 5‐year relative survival is also calculated for the whole period 2004–13 with 95% CIs.

Overall relative survival estimates were age‐standardized using Brenner's method.^25^ The reference age distribution was defined by the International Cancer Survival Standards (ICSS), with weights for specific PC age‐groups as follows: 0–54/19, 55–64/23, 65–74/29, 75–84/23, 85+/6.

The publically available Swedish quality register for PC was used to collect relevant diagnostic and treatment related background data on patients (https://statistik.incanet.se/npcr/). These data were separate from the other register data used and were not individually linked.

## 3 RESULTS

PC patient numbers are shown in age‐groups by stage in two periods, 2004–08 and 2009–13 (Table 1). T stages included only patients who were M0 (no metastasis) or Mx (metastasis status undefined). Case numbers between the periods increased for all other stages but T3 and T4; T1 increased from 18,889 to 22,460 patients. Case numbers increased in younger patient groups but decreased among patients diagnosed at age 75+ years. The largest age‐group was 65–74 years, and age‐group 85+ years accounted only for 5.56% of cases in the latter period. While M1 accounted for 7.70% of all patients in 2009–13, M1 patients at age 85+ accounted for 18.1%. The proportion of M1of all patients increased stepwise with age, from 4.3% in age‐group 15–64, 6.3% in age‐group 65–74 and 12.3% in age‐group 75–84. The proportions were somewhat higher in period 2004–08: 5.0%, 6.7%, 11.0%, 15.1%.

**TABLE 1:** Number of prostate cancers in Sweden by age at diagnosis and stage in two periods.

Stage	2004–2008					2009–2013				
	15–64	65–74	75–84	85+	Total	15–64	65–74	75–84	85+	Total
T1/M0Mx	7736 (40.96%)	7413 (39.25%)	3257 (17.24%)	483 (2.56%)	18,889 **(44.55%)**	8476 (37.74%)	10,213 (45.47%)	3318 (14.77%)	453 (2.02%)	22,460 **(48.15%)**
T2/M0Mx	3536 (28.19%)	4696 (37.44%)	3592 (28.64%)	719 (5.73%)	12,543 **(29.58%)**	3781 (27.96%)	5854 (43.30%)	3187 (23.57%)	699 (5.17%)	13,521 **(28.98%)**
T3/M0Mx	1097 (16.15%)	2173 (31.99%)	2644 (38.92%)	879 (12.94%)	6793 **(16.02%)**	1134 (17.81%)	2279 (35.80%)	2162 (33.96%)	791 (12.43%)	6366 **(13.65%)**
T4/M0Mx	92 (10.77%)	196 (22.95%)	359 (42.04%)	207 (24.24%)	854 **(2.01%)**	84 (11.81%)	187 (26.30%)	257 (36.15%)	183 (25.74%)	711 **(1.52%)**
M1	650 (19.56%)	1047 (31.51%)	1220 (36.71%)	406 (12.22%)	3323 **(7.84%)**	607 (16.90%)	1262 (35.14%)	1253 (34.89%)	469 (13.06%)	3591 **(7.70%)**
Total	13,111 (30.92%)	15,525 (36.61%)	11,072 (26.11%)	2694 (6.35%)	42,402 **(100.00%)**	14,082 (30.19%)	19,795 (42.43%)	10,177 (21.82%)	2595 (5.56%)	46,649 **(100.00%)**

*Note*: Row totals are bolded.

When reviewing case numbers for Mx in the above two periods, we noted that Mx was the main M classification of PC (71%) in 2004–08 while in 2009–13 it had almost disappeared (0.2%). The case numbers suggested that in the latter period Mx was converted to M0. We tested survival in M0 and Mx and indeed there were no large difference (Figure S1). This prompted us to combine the M0 and Mx codes.

### 3.1 Relative 1‐ and 5‐year survival

Relative 1‐year survival by stage (only T3, T4, and M1; for T4 only the two youngest age groups had enough cases) is shown in age‐groups in Figure S2 continuously from 2004 to 2017. T stages were all M0 or Mx. The older patients with T3 and M1 tumors and the youngest patients with T4 and M1 tumors appeared to gain in survival. A small decrease in survival was noted for T3 patients between 65 and 84 years.

**FIGURE 1:**
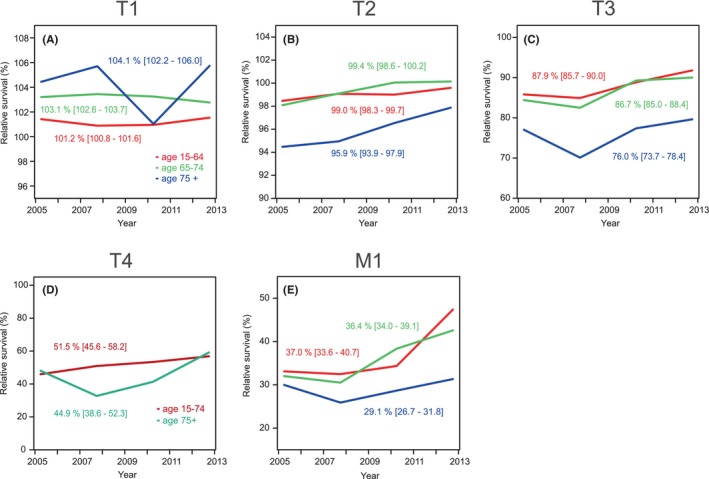
Relative 5‐year survival % in prostate cancer in Sweden from 2004 to 2013 by stage with 95% CI, T1 (A), T2 (B), T3 (C), T4 (D) and M1 (E). Age‐groups are 15–64, 65–74, 75+ years. Note the different scale in y axis in A and B from the other panels.

In Figure 1, 5‐year survival is shown in age‐groups for each stage. Because of the low number of patients, the two oldest age groups were combined (75–84 and 85+ years). For T1, all relative survival data were marginally over 100% **(**Figure 1A **).** For T2, survival for younger patients was 99.0% or higher and for the elderly patients it was 95.9% **(**Figure 1B **);** all curves showed an upward trend. For T3, survival decreased by age from 87.9%, to 86.7% and a significant drop for aged 75+ men, 76.0% (Figure 1C). For M1 patient the age group order was maintained 37.0%, 36.4%, and 29.1% but only the elderly showed a significantly worse survival with an apparent time‐dependent increase in the gap to survival of the younger men **(**Figure 1E **)**.

**FIGURE 2:**
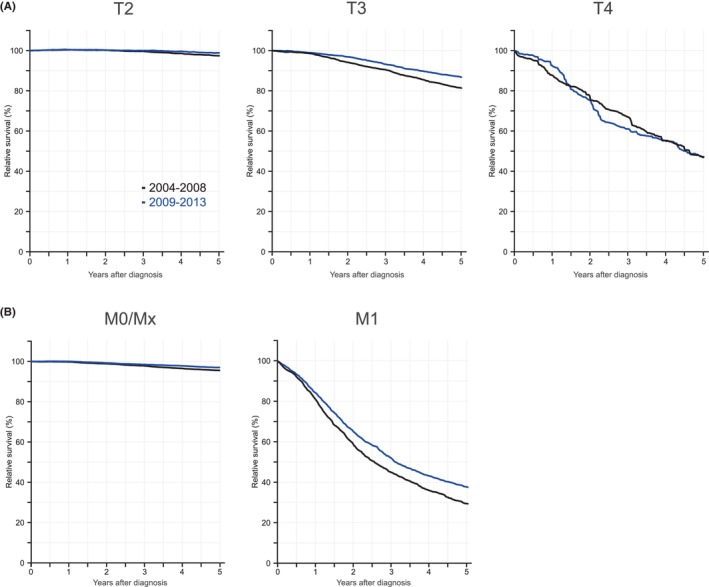
Relative annual survival trend in prostate cancer in Sweden by stage in two periods, 2004–2008 (lower curve) and 2009–2013 (upper curve), (A) T2, T3 and T4; (B), M0/Mx and M1. All T stages are M0 or Mx.

Relative 5‐year survival by stage is shown in Figure 2 in two periods, 2004–08 and 2009–13. T stages were all M0 or Mx. The upper panel (A) describes the gradually decreasing survival of patients from T2 to T4; for T2 (and T1, not shown) 5‐year survival was close to 100%. For T3 survival in the first period was 81.4% (79.0–83.8) and in the second one 86.7% (84.8–88.8). For T4 the difference was not significant. Fo M0/Mx survival in the first period was 95.5% (94.9–96.1) and in the second one 97.0% (96.4–97.5). For M1 survival in the first period was 29.4% (26.2–32.9) and in the second one 37.5% (34.1–41.3).

### 3.2 Conditional survival

**FIGURE 3:**
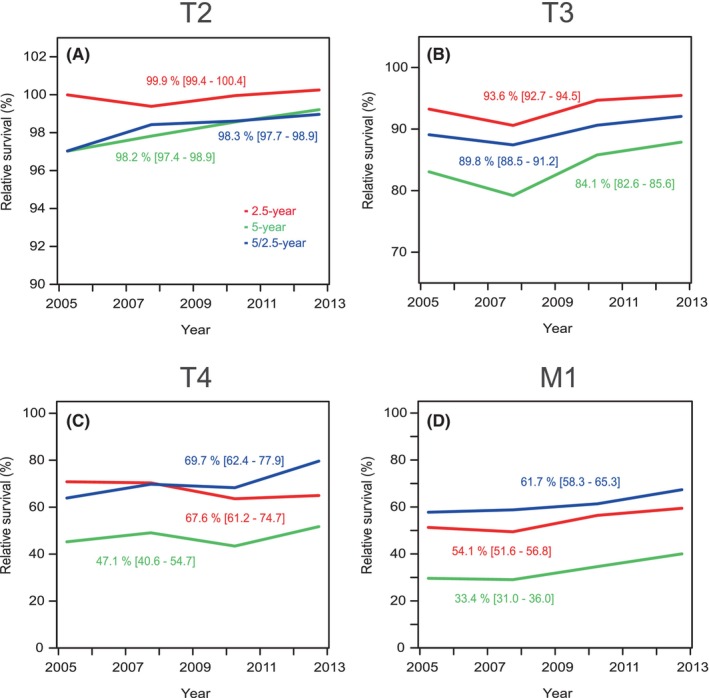
Relative 2.5‐, 5‐ and 5/2.5‐year survival % in prostate cancer in Sweden by stage with 95%CIs, T2 (A), T3 (B), T4 (C) and M1 (D). Note the different scale in y axis between A and B.

In Figure 3, we plot 2.5‐ and 5‐year relative survival together with conditional 5/2.5‐year relative survival (i.e., survival of living patients at years 2.5–5) to allow step‐wise assessment of survival up to years 2.5 and 5. In Figure 3A plots are shown for T2/M0Mx patients for which 2.5‐year survival was 100%, close to 2% units better than 5/2.5‐ and 5‐year survival (T1 is not shown because data were similar). In Figure 3B, T3/M0Mx patients, 2.5‐year survival was 93.6%, that for 5/2.5‐year survival was 89.8% and for 5‐year survival it was 84.1%, all with significant differences. In Figure 3C of T4 patient data, 5/2.5‐year survival (69.7%) has taken over the 2.5‐year survival (67.8%) and both were some 20% units better than the 5‐year survival (47.1%). In Figure 3D plots are shown for M1 patients, with 61.7% 5/2.5‐year survival, significantly better than 2.5‐year survival (54.1%) which in turn was significantly better than 5‐year survival (33.4%).

We show similar analysis for 1‐, 5‐ and 5/1‐year survival in Figure S3. The main feature is that 5‐ and 5/1‐year survival curves are close to each other and below 1‐year survival in T2 and T3 (A and B). In T4 and M1 5/1‐year survival was better than 5‐year survival but both were far below 1‐year survival (C and D).

**FIGURE 4:**
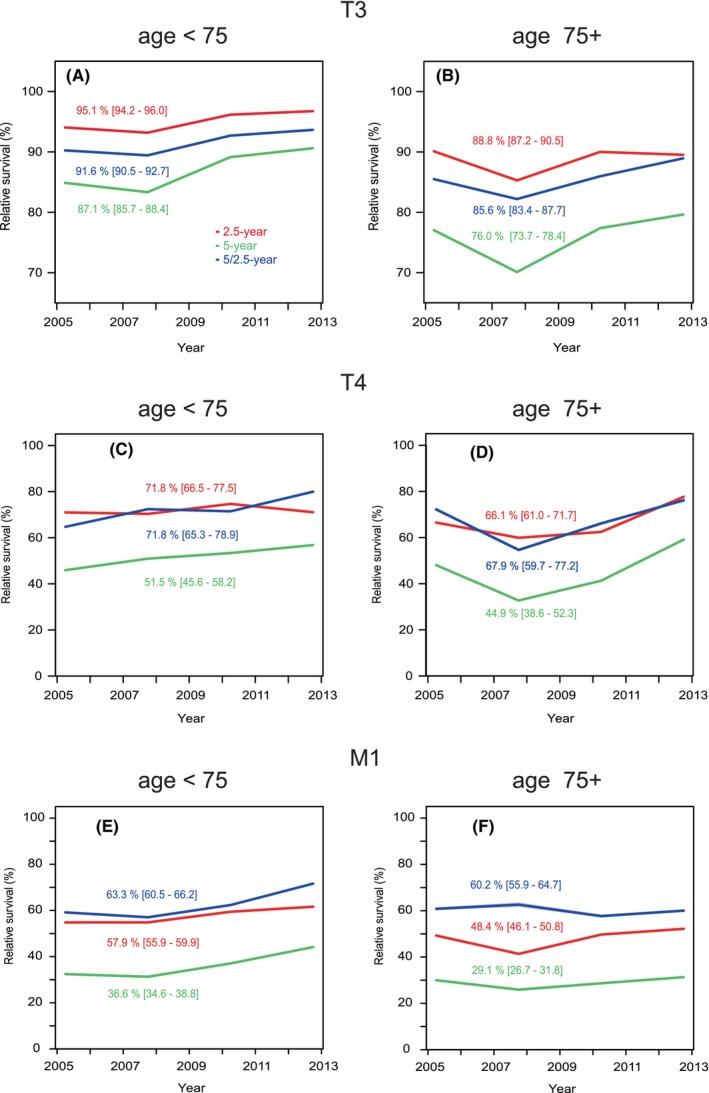
Relative 2.5‐, 5‐ and 5/2.5‐year survival % in prostate cancer in Sweden in stages T3 (A, B), T4 (C, D) and M1 (E), F9 in age‐groups <75 (on the left) and 75+ (on the right).

In Figure 4 data from Figure 3 are divided by age 75 years and only data for T3 to M1 are shown with largest difference between age‐groups. In Figure 4A,B, survival order is 2.5, 5/2.5, 5 year, and in 75+ old men 5%–10% units below younger men. In Figure 4C,D, one can observe the almost identical 2.5‐ and 5/2.5‐survival, about 20% units better than 5‐year survival. Panels E and F of M1 data show significant differences between all survival curves featuring suppression of 2.5‐year survival below 5/2.5‐year survival, with largest differences in the 75+ year patient population. The time tends show modest improvements for the three survival metrics in the <75 year population but not among the older patients.

Survival data are collected from the above results and summarized in Table 2.

**TABLE 2:** Summary of survival results up to year 2013.

Stage	Survival 1‐year, %	Survival 2.5‐year, %	Survival 5‐year, %
T2	100	100	98
T3	98	95	87
T4	90	64	48
M1	85	57	38

### 3.3 Clinical supplementary information

In Table S1 we have collected data from the publically available Swedish quality register for PC which may help understand the periodic changes and disadvantaged survival of the aged patients (https://statistik.incanet.se/npcr/). The diagnostics item 1 shows that the reason for diagnosing PC has become overwhelmingly through health controls but for men 80+ years lower urinary tract syndromes (LUTSs) were the main reason until 2016. Item 2 shows a diagram of risk classes, referred to in the Introduction. Additionally, we show data for men aged 80+ years, with distinct risk classes, dominated by regional and distant metastases, the proportions of which have remained high. Consequently, diagnostic PSA levels (item 3) were high among old men, concentration 20–99.9 ng/mL being the leading concentration interval. Magnetic resonance (MR) imaging was rarely used in the study period (2016–2018, all 11%, age 80+: 2%) (item 4). Bone imaging on the other hand gained momentum during the years with implications for the proportions of M0 or M1 patients (period of diagnosis: 2003 and 2018, all 48.9% and 80.3%, men at age 80+ 24.1% and 64.3%) (item 5).

In Table S1 related to treatment, the proportion of patients seen in multidisciplinary conferences in 2012–18 was 48%, and it was 22% for aged men (item 1). The proportion of high risk patients offered curative treatment has increased and in 2018 it reached 70.3% for all and 16.3% of aged men (item 2). Item 3 illustrates the trend for primary treatment strategy; the rate of surgery has remained constant around 25% with a slight decrease, while the amount of radiotherapy has increased from 13% in 2003 reaching surgery volumes (23%) in 2019. The other half have been managed noncuratively (conservative treatment/active surveillance/watchful waiting). Among aged men use of curative treatment has been minimal in the early time period of our study. Some 10% of elderly men received radiotherapy in the latter period while no surgery was performed. External radiation has been the main type of radiotherapy during our study period, while it was more often combined with brachytherapy in the early time points (25% in 2008 vs. 15% in 2018, item 4). Locally advanced tumors received more curative intent therapy in the latter period (2003: 23.7% vs. 2018: 62.5%, item 5). MR use in targeting radiotherapy increased from 31.1% in 2003 to 75.1% in 2018 (item 6). Long term (at least 18 months) adjuvant ADT treatment after radiotherapy for high‐risk patients increased from 8.6% in 2008 to 70.0% in 2018 (item 7). In the early time period 1/3 of the patients received bicautamide only while 2/3 where treated with GnRH agonist only or combined with bicalutamide. This changed gradually and around 2018, 90% received bicalutamide only (this is according to the Swedish guidelines while international guidelines generally recommend GnRH agonist, item 8). Over 90% of high‐risk patients received neoadjuvant ADT (2008 91.6% and 2018 94.6%, item 9). In the first time period half of the neoadjuvant treatments were administered with GnRH agonist only and half with GnRH agonist combined with bikalutamide. During the latter timeperiod of our study most neoadjuvant treatments were performed with the combination modality (reaching more than 2/3, item 10).

## 4 DISCUSSION

The present study covered the period from 2004 to 2018 which coincided with the culmination of PC incidence in Sweden after PSA introduction. This period has also seen an intense renewal of treatment of PC.^15^, ^18^, ^26^, ^27^ We could observe in the present stage‐specific analysis that relative survival increased in T3 and M1, patients with distal metastases (from 29.4% to 37.5%). As a novel finding we were able to define critical survival times depending on stage and age. Applying conditional relative survival up to 5 years we could show that for T3 and lower stages, the worst survival was between years 2.5 and 5. For T4 survival was worst at around year 2.5 and for M1 it was between years 1 and 2.5 (Figure 3). We could also observe the strong age‐group dependence of survival which has been reported before.^13^, ^14^ Even though active treatment of old patients has increased, our data showed that 5‐year survival in patients aged 75+ years only marginally improved compared to younger patients (Figure 1).

There has been a shift in the stage classification in PC and in our last covered 5‐year period almost half (48.2%) of all cases were T1. Compared to the previous 5‐year period, T1 was the only class that has increased; however, M1 decreased only marginally (from 7.8% to 7.7%). Unfortunately older patients are still relatively commonly diagnosed in the higher risk categories with high PSA levels (Table S1, diagnostics, item 1). According to Figure S3 relative 1‐year survival among all patients in T2 (identical for T1) was essentially 100%, it was 98% in T3 patients, in T4 patients it increased from below 90% to 94% and M1 patients it increased marginally to 84%. As T4 patients are less than 50% of M1 patients, most early deaths (up to year 1) were contributed to by M1. Relative 5‐year survival was close to 99% in T2, it was 84% in T3, it was 50% in T4 and 37% in M1. A clear separation of 5/1‐and 5‐year survival was noted only in T4 and M1 which can be explained by early deaths in these stages compared to T2.

We observed a systematic age dependent increase in the proportion of M1 of all PC, increasing from 4.3% in age‐group 15–64 years to 18.1% of patients at age 85+ years in period 2009–13. The proportions were similar also in the preceding period 2004–08. This is probably related to the reasons which lead to diagnosis of PC, being health examinations in the younger patients and LUTS and other symptoms in the older ones. Also the proportion of high‐risk patients undergoing bone imaging increased during the study period from 48.9% in 2003 to 80.3% in 2018 (Table S1, diagnostics/item 5). Similar findings were reported in the comparison of the US and the Danish metastatic PC rates over time, and a large reduction in the proportion of metastatic PC occurred in the USA during the introduction of PSA testing.^28^ However, the reasons may be more complicated. In the Netherlands, metastatic data are available on all common cancers and the proportion of M1 of all PC varies extensively over time.^29^ It was over 25% around 1990 and then decreased to 13% around 2010 and increased again towards 2018 to almost 20%.^29^ PSA testing for PC started in the Netherlands at around 1990 similar to Sweden.^30^ In the Netherlands relative 1‐year survival of M1 patients increased from 79% in 1989–93 to 89% in 2014–18, and 5‐year relative survival increased from 23% to 42%.^29^ Our data for M1 in 2009–13 were 83.8% for 1‐year (Figure S3) and 37.5% for 5‐year relative survival (Figure 3), thus somewhat below the Dutch survival figures although our data were from earlier years. In Norway, the proportion of M1 is similar to Sweden, 7%, and median survival was 2.3 years in 2004–09 increasing to 3.3 years in 2015–18.^14^ The median survival in 2010–15 was 2.7 years according to a previous Swedish study and our current median survival for 2009–13 is 3.1 years.^13^ The large difference in the proportion of M1 in Sweden/Norway and the Netherlands may raise questions about diagnostic criteria; further, does the better survival in a country with higher proportions of M1 suggest that relatively more benign forms of metastatic tumors were included?

Sweden has national guidelines for diagnostics and treatment of PC which are periodically revised considering the European Society for Medical Oncology (ESMO) and European Association for Urology (EAU) guidelines.^12^, ^15^, ^31^, ^32^ The guidelines recommend risk/stage adaptive therapies and diagnostics. Active surveillance is preferred in less aggressive PC, while more aggressive local cancers should typically be treated with prostatectomy or radiotherapy.^15^, ^31^ In more advanced states ADT and chemotherapy should be applied with addition of novel agents in castration resistant cases.^15^, ^31^ The Swedish guidelines differ from the European ones in a few aspects, such as recommending bicalutamide monotherapy instead of GnRH agonists for adjuvant therapy after curative intent radiotherapy in intermediate/high‐risk patients.^12^, ^32^ Comparison of the Swedish clinical outcomes in PC with other countries should consider differences in treatment.

Most guidelines advocate “watchful waiting” for patients who are expected to live less than 10 years instead of active surveillance (with e.g., PSA follow‐up) for PC.^31^, ^33^ The main reasons to this recommendation include risk of biopsy and risk of curative treatment related adverse events. It has been proposed that salvage treatment with ADT for the elderly (life expectancy less than 10 years) who advance to symptomatic or metastatic disease lead to optimal results.^31^, ^33^ The traditional transrectal prostate biopsy results in infections, including sepsis for a few percent of patients and especially for the frail this can be lethal. Mainly for this reason, transperineal biopsies as the primary biopsy method have been recently recommended by several guidelines including the EAU.^31^ Additionally, surgery has become more common and safe for the elderly and radiation more precise and tolerable. According to our results and results presented by others, the previously used 10‐year life expectancy limit might need reevaluation in the transperitoneal biopsy era.

In Table S1 we collected data on the practical implementation of such guidelines, which in the diagnostics part showed an increasing proportion of intermediate‐risk cancers and lower proportions of any higher‐risk cancers, although the proportion of distant metastases (12%) remained constant since 2005 and remained high in patients aged 80+ years. In the treatment part, the national register data show a trend change to active treatment since 2006 which however has enticed few old patients; even in 2018 only 16.3% of men at age 80+ years received curative primary treatment. In locally advanced PC, radical radiotherapy or prostatectomy increased from 15% to 43% of patients in 15 years.^13^ The national register does not include data on medical treatment, except for documentation that androgen blockade as adjuvant and neoadjuvant treatment in radiotherapy for high‐risk PC has become a common practice. Traditionally ADT has been the basis of treatment for metastatic PC and additional treatment options emerged into the new millennium, including first docetaxel and later enzalutamide, abiraterone and radiotherapy with 223Ra.^13^, ^14^

In conclusion, we showed survival trends around the culmination of the incidence changes which had started with the introduction of PSA testing. We could show small improvements in stage‐and age‐related survival up to year 2017 for 1‐year and to year 2013 for 5‐year survival. Applying conditional relative survival, we showed that the most critical survival period for T1 to T3 PC was after 2.5 years of diagnosis, for T4 it was at about 2.5 years and for M1 it was between years 1 and 2.5 after diagnosis. Such data can be used in devising follow‐up schemes for PC patients and further specifications can be introduced for T1 to T3 when extending conditional survival times past 5 years. Despite commonly available PSA testing, many patients are still diagnosed at high‐risk or advanced‐state calling for more effective methods of early detection, this might be achieved by properly conducted population wide screening.^34^ The proportions of high risk and M1 disease in 80+ year old patients remain very high. Many of these men are in good condition and may live another 10 years (mean over 8 years, https://knoema.com/atlas/Sweden/topics/Demographics/Age/Life‐expectancy‐at‐age‐80‐years). Slow improvement in 5‐year survival of elderly compared to younger patients at stage T3 and higher may suggest undermanagement.

## AUTHOR CONTRIBUTIONS

**Kari Hemminki:** Conceptualization (lead); project administration (lead); supervision (equal); writing – original draft (equal). **Frantisek Zitricky:** Formal analysis (lead); writing – review and editing (equal). **Kristina Sundquist:** Data curation (equal); resources (equal); writing – review and editing (equal). **Jan Sundquist:** Data curation (equal); resources (equal); writing – review and editing (equal). **Asta Försti:** Data curation (equal); validation (equal); writing – review and editing (equal). **Akseli Hemminki:** Conceptualization (supporting); investigation (equal); writing – original draft (supporting); writing – review and editing (equal). **Otto Hemminki:** Conceptualization (supporting); investigation (supporting); resources (supporting); writing – original draft (supporting); writing – review and editing (equal).

## FUNDING INFORMATION

Supported by the European Union's Horizon 2020 research and innovation programme, grant No 856620, The Swedish Research Council, Jane and Aatos Erkko Foundation, Sigrid Juselius Foundation, Finnish Cancer Organizations, University of Helsinki, Helsinki University Central Hospital, Novo Nordisk Foundation, Päivikki and Sakari Sohlberg Foundation, the Cooperatio Program, research area SURG, National Institute for Cancer Research—NICR (Programme EXCELES, ID Project No. LX22NPO5102), funded by the European Union—Next Generation EU and the SALVAGE project, reg.no: CZ.02.01.01/00/22_008/0004644.

## CONFLICT OF INTEREST STATEMENT

A.H. is shareholder in Targovax ASA. A.H. is employee and shareholder in TILT Biotherapeutics Ltd. Other authors declared no conflict of interest.

## ETHICS STATEMENT

The study was approved by the Regional Ethical Review Board in Lund University, February 6, 2013 (Reference 2012/795 and subsequent amendments). The Regional Ethical Review Board in Lund University waved the need to include informed consent. The study was conducted in accordance to Declaration of Helsinki.

## Supporting information


Figure S1.

## Data Availability

The source of the used original data is the Swedish Board of Health and Welfare, which needs to be contacted by anyone wanting to access the data.
