# Supplementary material for: Critical survival periods in prostate cancer in Sweden explored by conditional survival analysis
Source: Cancer Med. 2024 Mar 28;13(7):e7126. doi: 10.1002/cam4.7126 (PMC10974700; doi:10.1002/cam4.7126)
Supplement: Supplementary file 1 — Figure S1. [file CAM4-13-e7126-s001.docx]

**Supplementary Table 1. Selected diagnostic and treatment data from the Swedish quality register for prostate cancer considering periods and age groups**

The years selected were 2003 and 2018, (if data were available, otherwise years available in that interval). Figures could not be modified and some included the latest year of 2023.

**DIAGNOSTICS**

**1. Principal reason for discovery of prostate cancer from 2004 to 2022, all patients (top), age 80+ (bottom).**


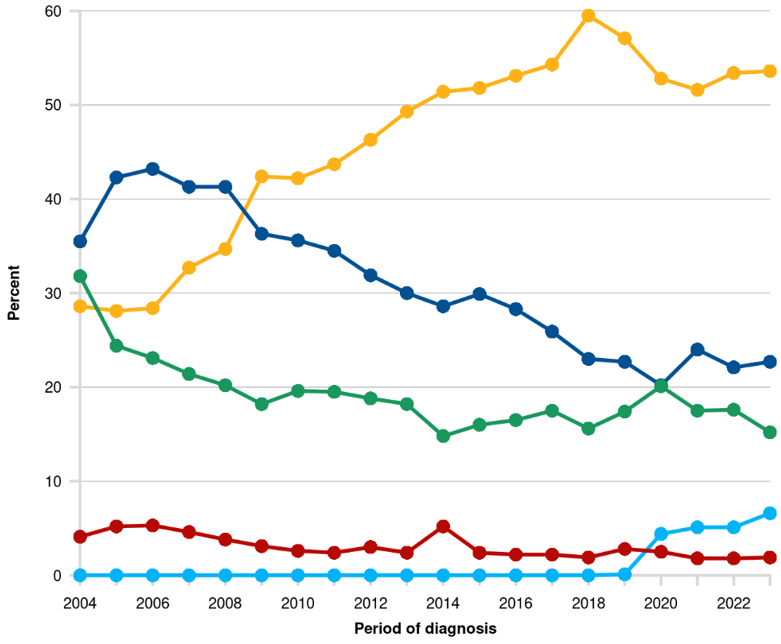

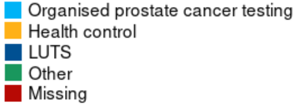


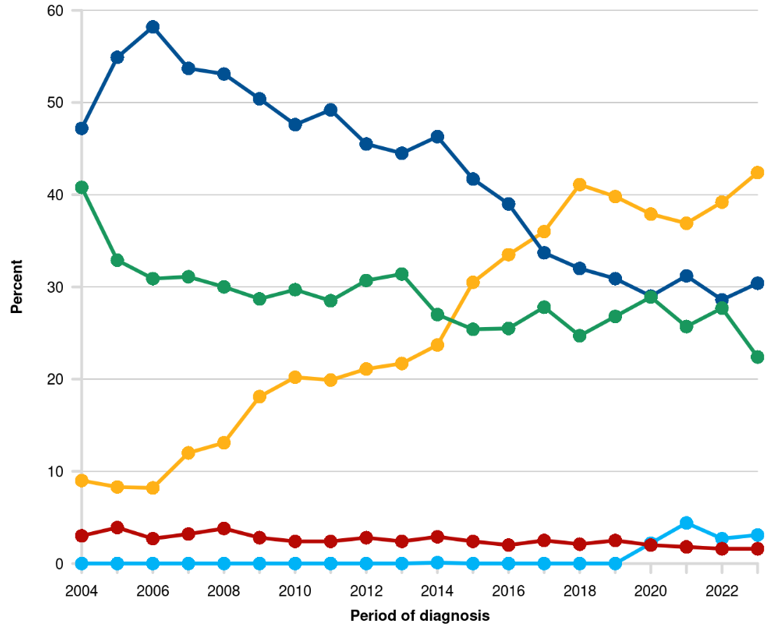


**Period of diagnosis: 2004 and 2018.**

**a) Elevated PSA-level at a health check up of a man with no symptoms, b) investigation of lower urinary tract symptoms (LUTS) or c) other symptoms, e.g. skeletal pain resulting from skeletal metastasis or blood in the urine (missing % not shown).**

2004: 28.6%, 35.5%, 31.8%. Men at age 80+: 9.0%, 47.2%, 40.8%.

2018: 55.9%, 23.0%, 15.7%. Men at age 80+: 41.2%, 32.0%, 24.8%.

**2. Distribution of risk classification for men with prostate cancer low-intermediate-high-regional met-distant met (for exact definition see Introduction of the paper), all (top) and age 80+ (bottom).**


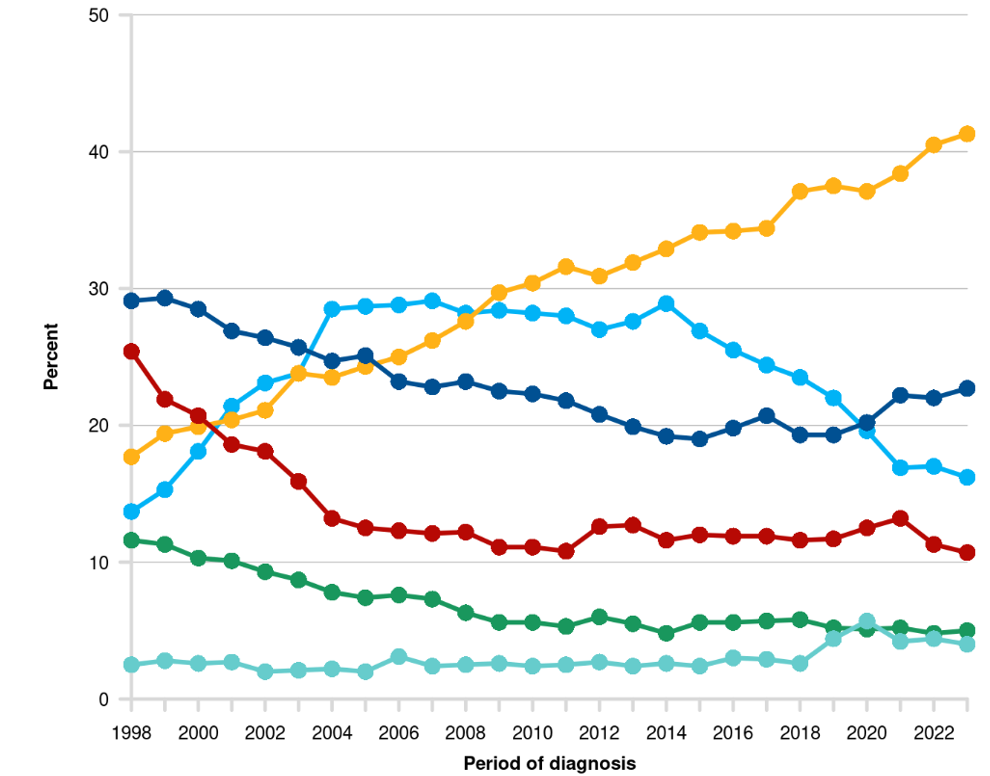

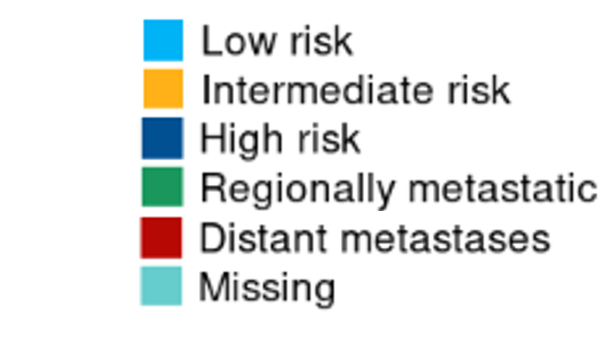


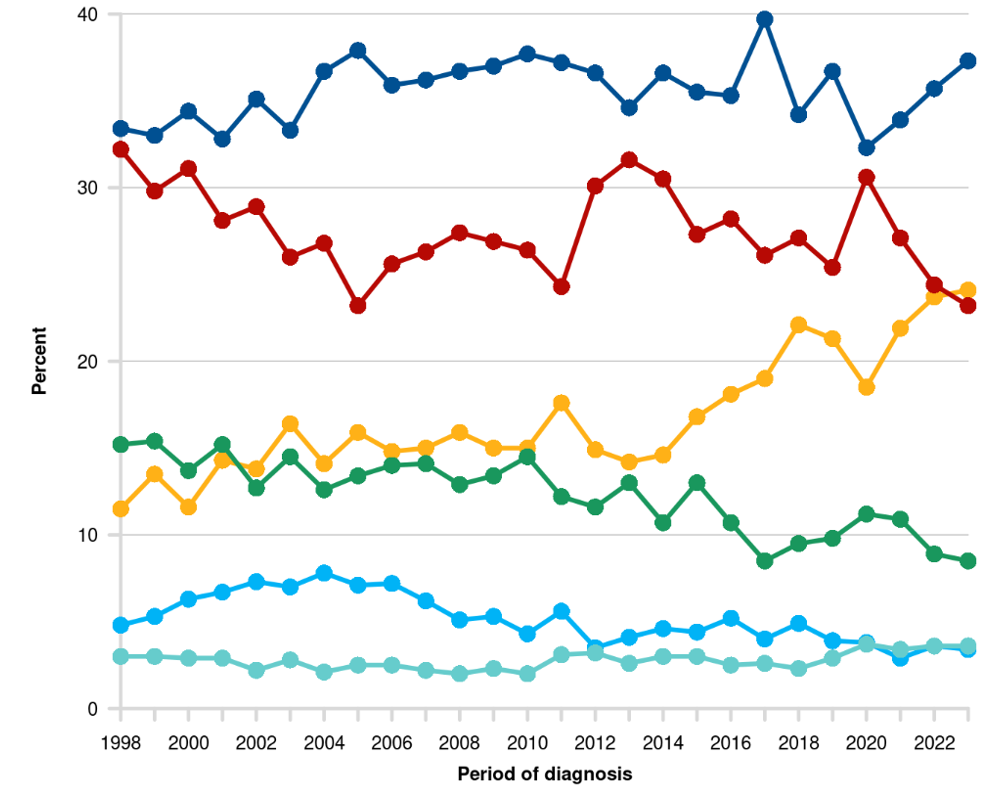


2003: 23.8-23.8-25.7- 8.7-15.9%. Men at age 80+: 7.0-16.4-33.3-14.5-26.0%

2018: 23.6-37.1-19.3-5.8-11.6%. Men at age 80+: 4.9-22.1-34.3- 9.5-27.1%

**3. Serum PSA-level (ng/mL) at time of PC diagnosis from 1996 to 2022 for all men (top) and men at age 80+ (bottom).**


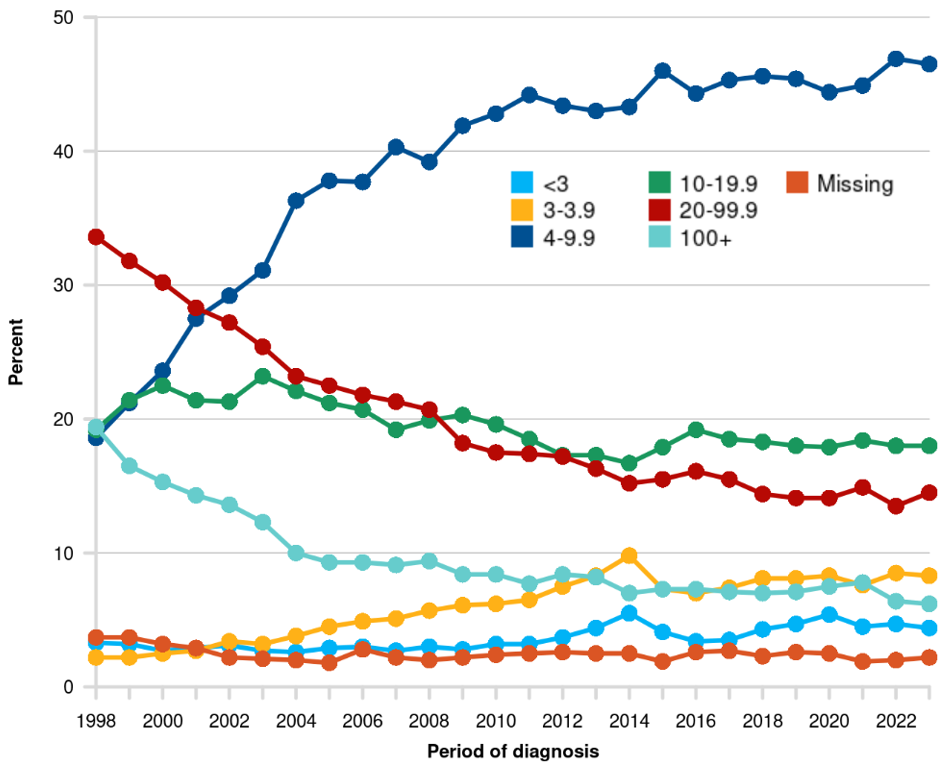


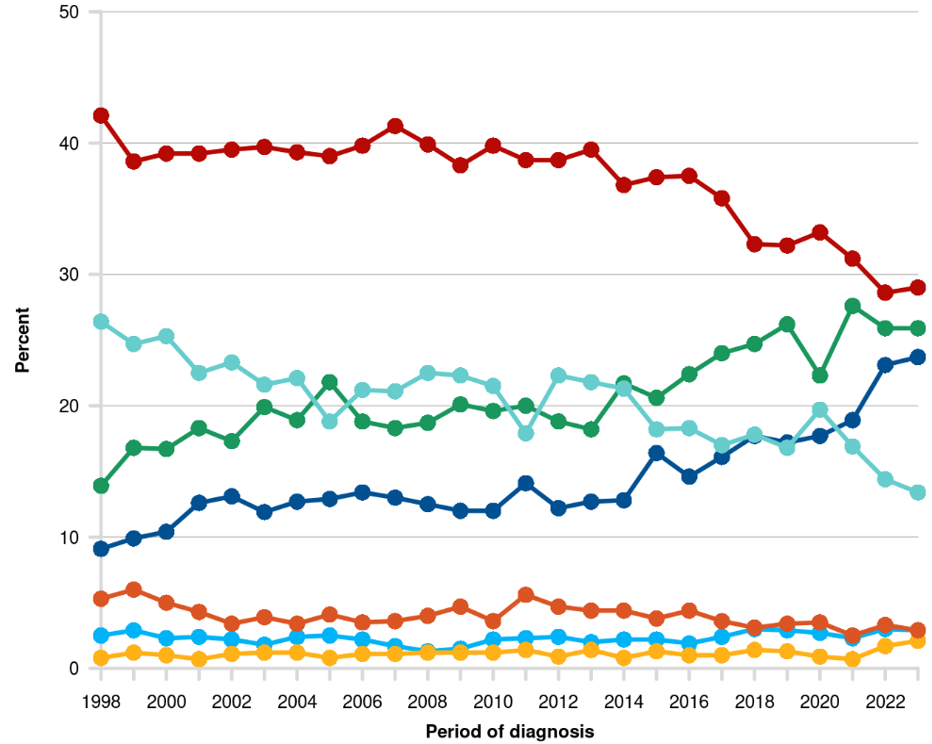


**4. MR performed before diagnostic biopsy among men with PSA < 100 ng/mL**

**Among men who underwent biopsy.**

Period of diagnosis: 2016-2018, all 11%, age 80+: 2%.

**5. Proportion of men with high risk prostate cancer that underwent bone imaging**

Period of diagnosis: 2003 and 2018, all 48.9% and 80.3%. Men at age 80+: 24.1% and 64.3%.

**TREATMENT**

**1. Multidisciplinary conference/reception for men at risk group at diagnosis: localized high risk/ locally advanced.**

Period of diagnosis: 2012-2018: all 48%, patients 80+ 22%.

**2. Proportion of men with prostate cancer given curative primary treatment**

Period of diagnosis: 2003 and 2018. Risk group at diagnosis: localized high. All 31.9% and 70.3%. Men at age 80+: 0.3% and 16.3%.

**3. Primary treatment strategy, per treating unit, all (top) and men aged 80+ (bottom)**


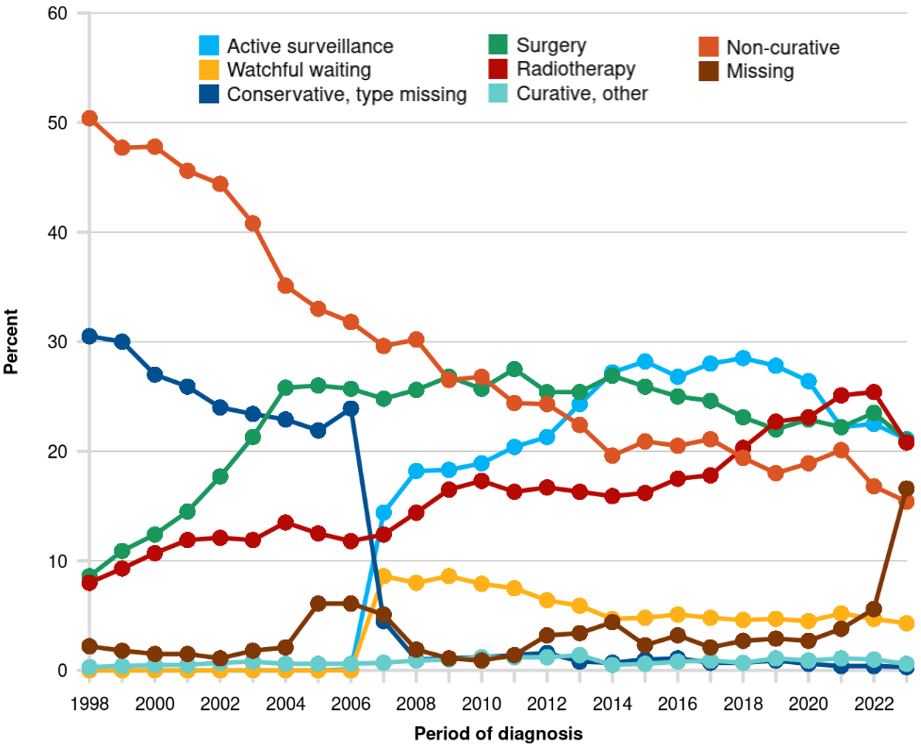


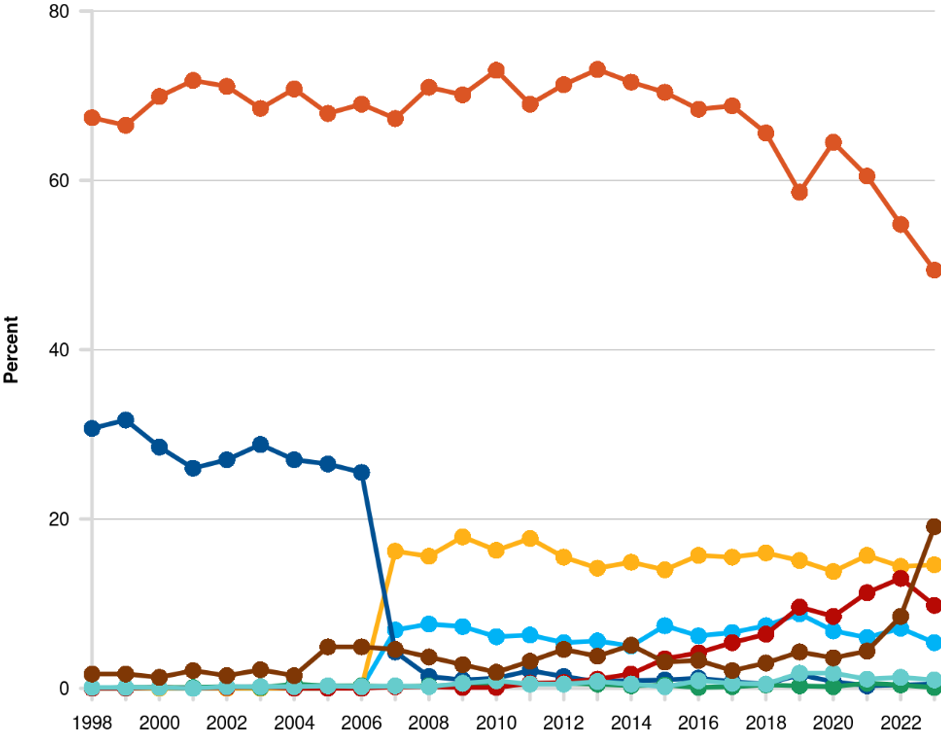


**4. Type of radiotherapy**


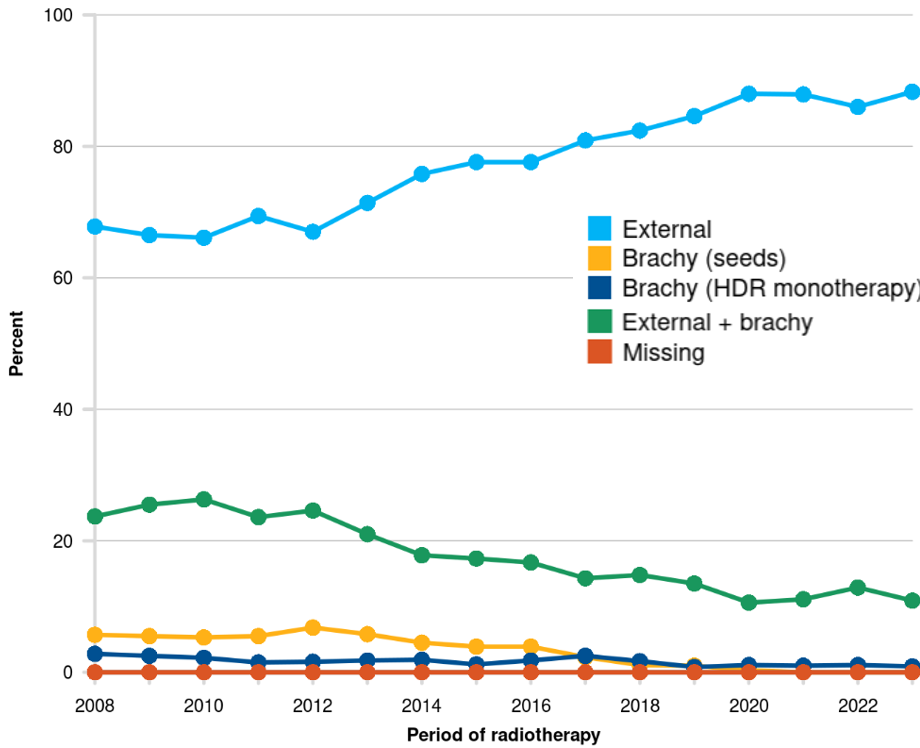


**5. Proportion curative radiotherapy or inclusion in SPCG-15**

**Risk group at diagnosis: Locally advanced* (Locally advanced: T3, N0/NX, M0 and PSA < 100 ng/ml). Age at diagnosis: 0-80.**

Period of radiotherapy: 2003 and 2018,l 2003: 23.7% 2018: 62.5%

**6. Proportion of external/external + brachy RT where MR was used in target definition**

Period of radiotherapy: 2008-2018.2008: 31.1% 2018: 75.1%.

**7. At least 18 months adjuvant ADT with antiandrogens after RT**

**Risk group at radiotherapy: Localised high risk* / Locally advanced**. Age at radiotherapy: 0-75.**

Period of radiotherapy: 2008-2018. 2008: 8.6% 2018: 70.0%.

**8. Adjuvant androgen deprivation therapy (ADT) is administered after radiotherapy in order to reduce the risk of tumour recurrence. Adjuvant ADT usually consists of bicalutamide, an anti-androgen drug, alternatively an GnRH-agonist injection can be used. The national prostate cancer care program recommends bicalutamide as adjuvant ADT. TAB total androgen blocking agent.**

**
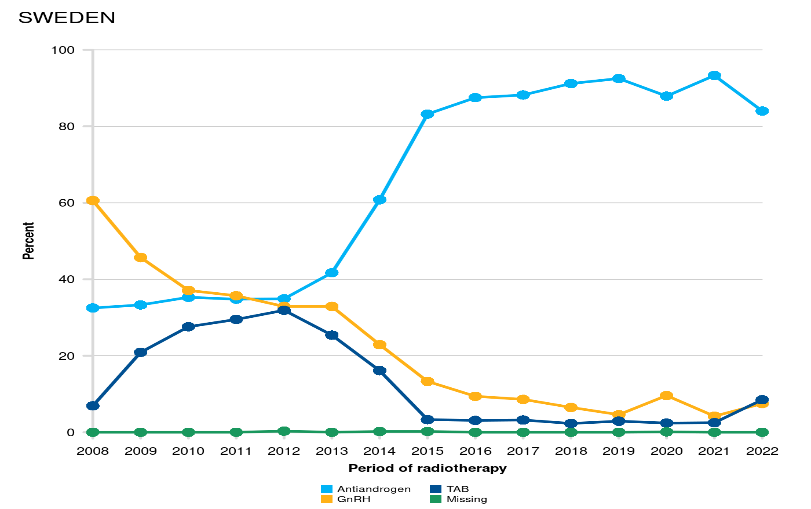
**

**9. Proportion given neoadjuvant ADT before RT, Risk group at treatment: High risk.**

Period of radiotherapy: 2008 and 2018: 91.6% and 94.6%.

**10. Type of neoadjuvant androgen deprivation therapy (ADT). Neoadjuvant androgen deprivation therapy (ADT) is administered before radiotherapy in order to increase tissue sensitivity to radiation. ADT can be administered in the form of GnRH-agonists or bicalutamide, an anti-androgen drug, or both substances simultaneously (TAB; total androgen blockade).**


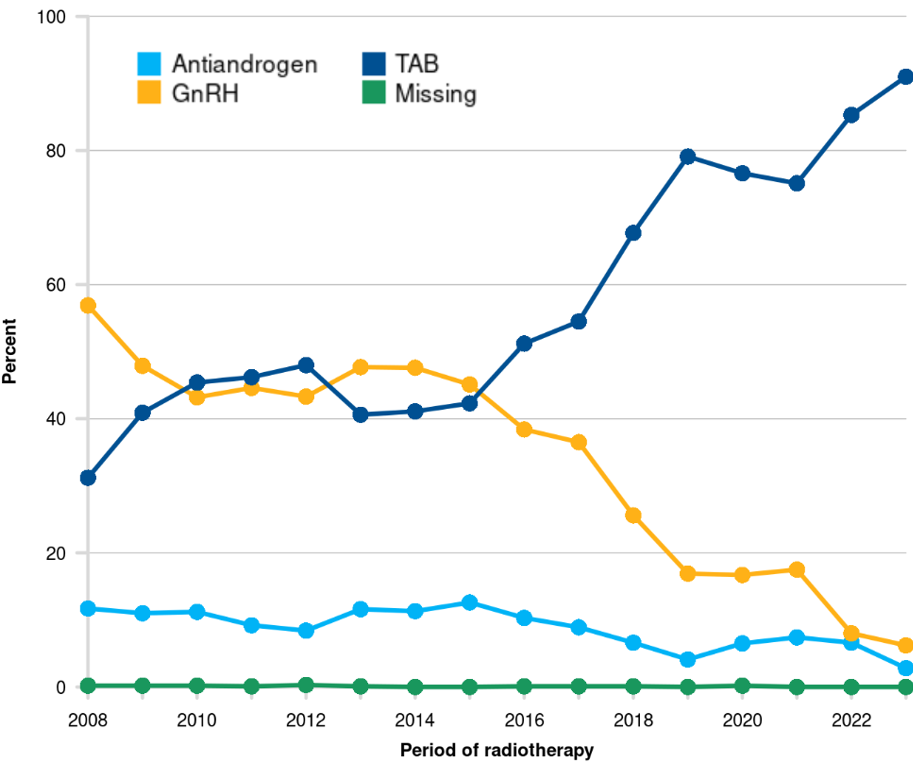


**
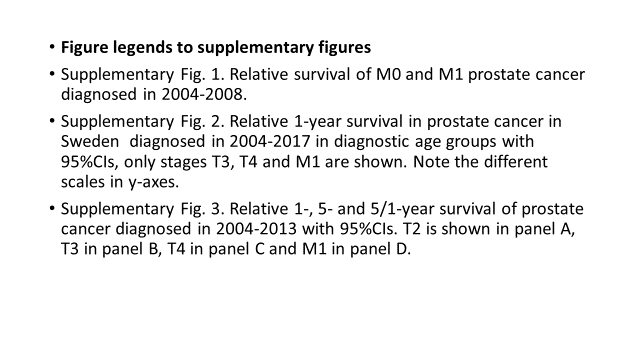
**

**
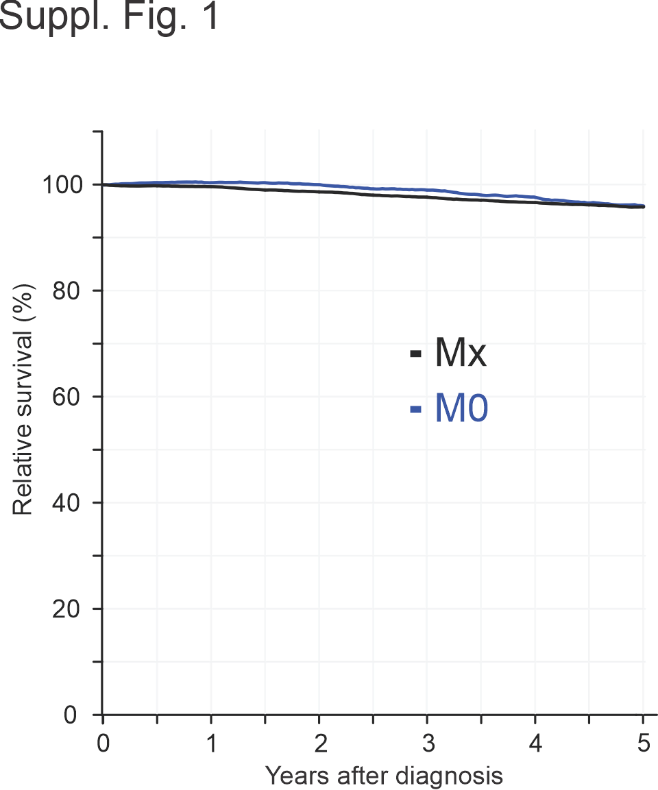
**

**
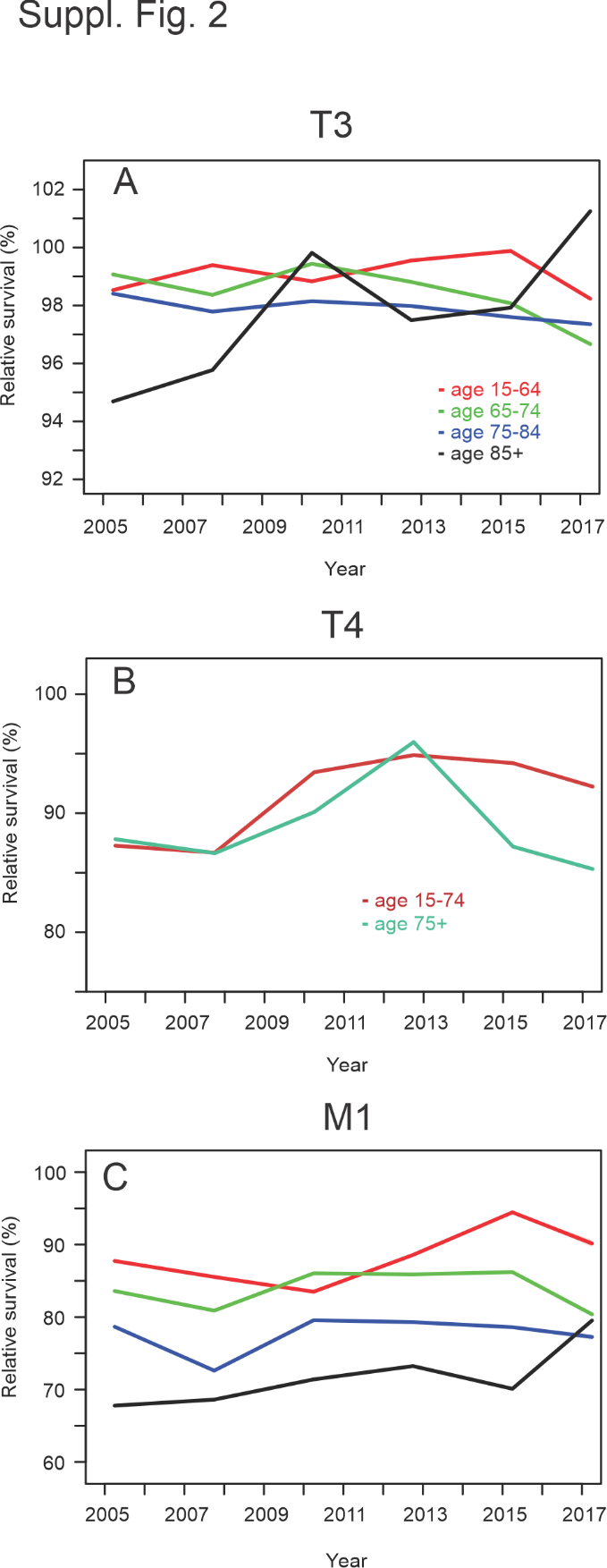
**

**
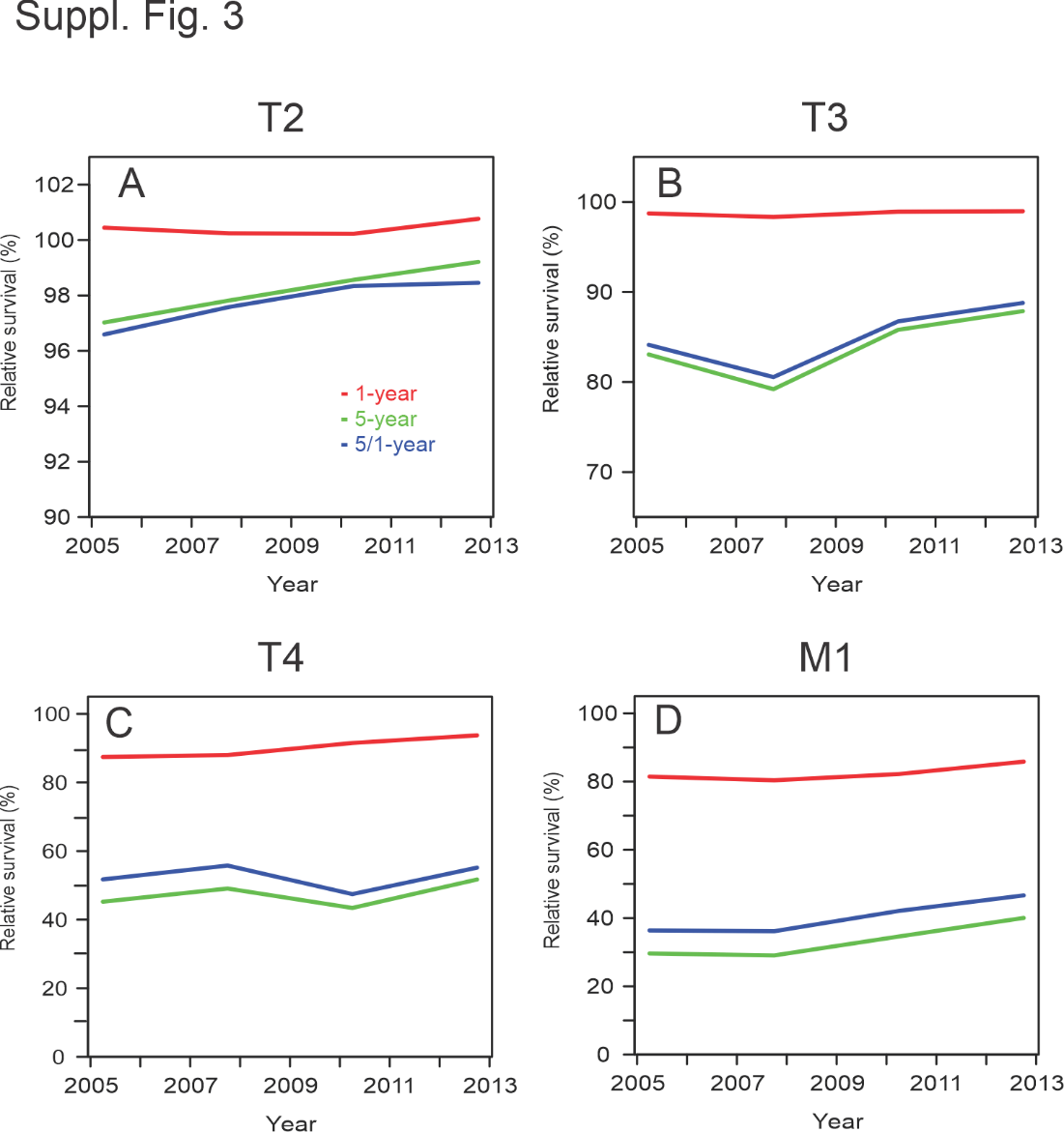
**
